# Genetic background influences the 5XFAD Alzheimer's disease mouse model brain proteome

**DOI:** 10.3389/fnagi.2023.1239116

**Published:** 2023-10-12

**Authors:** Cheyenne D. Hurst, Amy R. Dunn, Eric B. Dammer, Duc M. Duong, Sarah M. Shapley, Nicholas T. Seyfried, Catherine C. Kaczorowski, Erik C. B. Johnson

**Affiliations:** ^1^Goizueta Alzheimer's Disease Research Center, Emory University School of Medicine, Atlanta, GA, United States; ^2^Department of Biochemistry, Emory University School of Medicine, Atlanta, GA, United States; ^3^Department of Mammalian Genetics, The Jackson Laboratory, Bar Harbor, ME, United States; ^4^Department of Neurology, Emory University School of Medicine, Atlanta, GA, United States; ^5^Department of Neurology, The University of Michigan, Ann Arbor, MI, United States

**Keywords:** Alzheimer's disease, mouse models, proteomics, 5XFAD, B6xD2, translational, protein network analysis

## Abstract

There is an urgent need to improve the translational validity of Alzheimer's disease (AD) mouse models. Introducing genetic background diversity in AD mouse models has been proposed as a way to increase validity and enable the discovery of previously uncharacterized genetic contributions to AD susceptibility or resilience. However, the extent to which genetic background influences the mouse brain proteome and its perturbation in AD mouse models is unknown. In this study, we crossed the 5XFAD AD mouse model on a C57BL/6J (B6) inbred background with the DBA/2J (D2) inbred background and analyzed the effects of genetic background variation on the brain proteome in F1 progeny. Both genetic background and 5XFAD transgene insertion strongly affected protein variance in the hippocampus and cortex (*n* = 3,368 proteins). Protein co-expression network analysis identified 16 modules of highly co-expressed proteins common across the hippocampus and cortex in 5XFAD and non-transgenic mice. Among the modules strongly influenced by genetic background were those related to small molecule metabolism and ion transport. Modules strongly influenced by the 5XFAD transgene were related to lysosome/stress responses and neuronal synapse/signaling. The modules with the strongest relationship to human disease—neuronal synapse/signaling and lysosome/stress response—were not significantly influenced by genetic background. However, other modules in 5XFAD that were related to human disease, such as GABA synaptic signaling and mitochondrial membrane modules, were influenced by genetic background. Most disease-related modules were more strongly correlated with AD genotype in the hippocampus compared with the cortex. Our findings suggest that the genetic diversity introduced by crossing B6 and D2 inbred backgrounds influences proteomic changes related to disease in the 5XFAD model, and that proteomic analysis of other genetic backgrounds in transgenic and knock-in AD mouse models is warranted to capture the full range of molecular heterogeneity in genetically diverse models of AD.

## Introduction

Alzheimer's disease (AD) is the leading cause of dementia worldwide, with limited treatment options currently available. Clinical trials of potential disease-altering therapies for AD have had very high failure rates. Evaluation of failings at the preclinical stage has implicated poor translation between widely used mouse models of AD and human disease as a potential explanation (Cummings, 2018). Murine models of AD represent a cornerstone of disease research and have played a critical role in the preclinical development process. Traditional modeling of AD in mice has primarily focused on the overexpression of the amyloid precursor protein (APP) and presenilins 1 and 2 (PSEN1/2) that harbor familial AD (FAD) mutations. Such transgenic FAD models robustly develop β-amyloidosis and have been instrumental in understanding disease mechanisms and progression (Jankowsky and Zheng, 2017). However, no single model fully recapitulates the complex pathobiology of human disease, leading to gaps in cross-species translation. The need to develop and improve the validity and translatability of AD mouse models remains an ongoing challenge.

Mouse models of AD have been almost exclusively developed on a single inbred strain, the C57BL/6J (B6). The selection of B6 as the ideal background for developing AD model systems has been largely due to their desirable behavioral characteristics and the development of cognitive impairment and amyloid deposition (Lassalle et al., 2008; Sultana et al., 2019; Forner et al., 2021; Ammassari-Teule, 2022). For instance, the commonly used 5XFAD transgenic model was developed on the B6 background to overcome variable phenotypes produced on a hybrid background (Oblak et al., 2021). However, the utility of inbred strains has come under question in recent years as research has indicated the lack of generalizability and often opposing phenotypes across different strains expressing FAD mutations. Outcome measures of interest to AD researchers, such as learning and memory-specific task performance and amyloid pathology, are significantly impacted by mouse genetic background (Lehman et al., 2003; Ryman et al., 2008; Sittig et al., 2016).

Genetics contributes strongly to susceptibility and person-to-person heterogeneity for both familial and sporadic forms of AD. Twin studies have estimated the heritability of AD to be approximately 60–80% (Gatz et al., 2006). Even among autosomal dominant cases, the onset of clinical symptoms can vary across families that carry the same mutation (Bekris et al., 2010). Together, these findings highlight the complex interaction between genetic factors and phenotype in human AD and indicate that there are undefined genetic factors that contribute to disease risk or resilience. The complex relationship between genetic composition and phenotypic variability observed in human populations, which is lacking within genetically homogenous inbred mouse strains, has led to the hypothesis that incorporating genetic variability in AD mouse models could improve translatability. In support of this hypothesis, an AD transgenic mouse reference panel (AD-BXD) was recently developed to explore the impact of genetic complexity on phenotypic segregation and translation with human disease features (Neuner et al., 2019). The reference panel was generated by crossing 5XFAD mice on a B6 background with the BXD recombinant inbred strain series derived from B6 and DBA/2J (D2) crosses. The results from this study identified improved AD phenotypes relating to varied age of onset and rates of memory impairment across the resulting strains. These findings indicate a high potential value for incorporating genetic variability as a means to improve the face validity of transgenic mice. However, it is currently unknown how such genetic diversity influences protein changes related to disease in such models.

Quantitative proteomics has contributed to an improved understanding of human brain pathophysiology in AD (Rayaprolu et al., 2021; Johnson et al., 2022). In this study, we asked how genetic diversity introduced by crossing B6 and D2 mice (B6xD2), two fully inbred strains, affects the mouse brain proteome and its perturbation by the 5XFAD transgene. We found strong proteomic alterations related to both genetic background and transgene expression. We then integrated human brain proteomic data to assess relevant overlap and cross-species relationships. Our findings serve as a resource for the continued development of AD mouse models with improved translational power.

## Results

### Genetic background and AD genotype strongly contribute to proteomic variance

**Figure 1:**
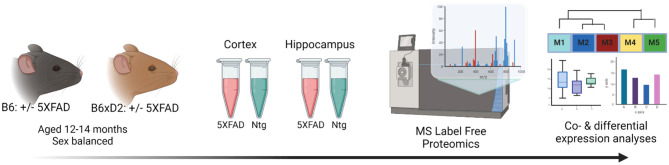
Study overview. Schematic representation of the experimental workflow for matched mouse brain tissue samples from the cortex and hippocampus from 5XFAD and non-transgenic (Ntg) mice on B6 (*n* = 10 5XFAD, *n* = 10 Ntg) or B6xD2 (*n* = 10 5XFAD, *n* = 10 Ntg) genetic backgrounds (*n* = 40 total per region). Tissue samples underwent enzymatic digestion and label-free mass spectrometry analysis, followed by co- and differential expression analyses.

To address how genetic variation in AD mouse model systems impacts the brain proteome, label-free quantitation mass spectrometry (LFQ-MS) analysis was performed on brain tissue from two brain regions (cortex and hippocampus) from 5XFAD transgenic and non-transgenic (Ntg) B6 and B6xD2 mouse lines as previously described (Johnson et al., 2020). The cortex and hippocampus are both vulnerable in AD and represent relevant regions of interest for proteomic analysis. Mice (*n* = 10 B6 5XFAD, *n* = 10 B6xD2 5XFAD, *n* = 10 B6 Ntg, *n* = 10 B6xD2 Ntg; five males, five females per group) were aged 13–14 months, a time at which significant pathology has developed in the 5XFAD model (Oakley et al., 2006; Oblak et al., 2021). Protein abundance data were pre-processed to remove proteins with higher missing values and sample outliers, resulting in a final data matrix of 3,368 proteins measured across 70 total samples (Figure 1, Supplementary Tables 1, 2). The term “line” will be used throughout to refer to the mouse genetic background (i.e., B6 or B6xD2), while “AD genotype” is used to denote the transgenic manipulation of the recipient mouse (Ntg vs. 5XFAD).

**Figure 2:**
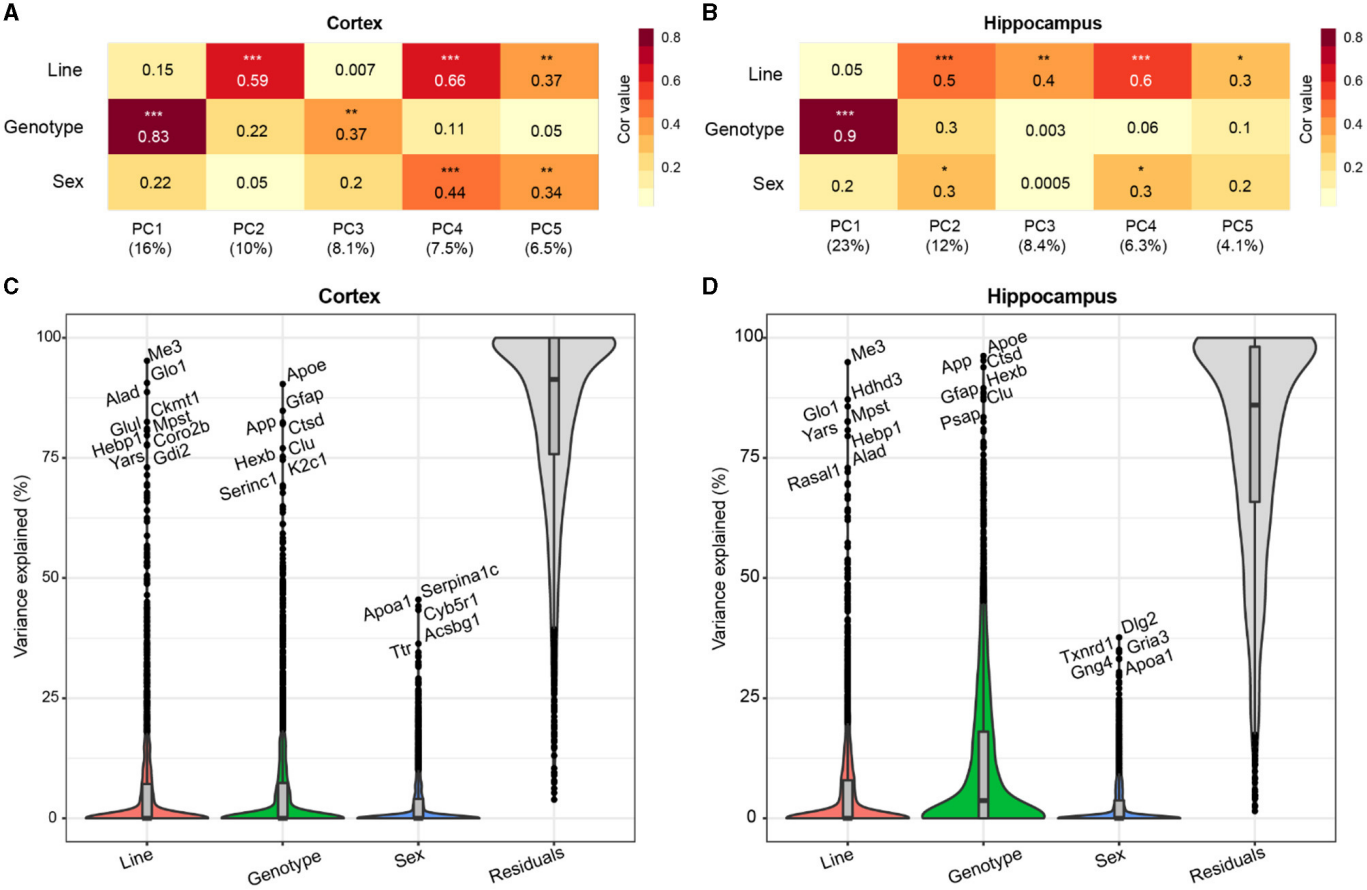
Genetic background and AD genotype strongly contribute to proteomic variance. **(A, B)** Principal component analysis (PCA) in the cortex **(A)** and hippocampus **(B)** was used to examine the relationship between the top five PCs and line, AD genotype, and sex traits using Spearman's rho correlation. Heatmaps represent the strength of correlation between traits and PCs (darker color indicates increasing strength of correlation). AD genotype was the most strongly associated trait with PC1 in the cortex (ρ = 0.83) and hippocampus (ρ = 0.9). *P* ^*^ <0.05, ^**^ <0.01, ^***^ <0.001. **(C, D)** Variance partition analysis was performed for the cortex **(C)** and hippocampus **(D)** to quantify the overall variance explained by each trait. Line and AD genotypes contributed to the highest percent variance explainable in both brain regions.

To assess the primary contributing features of variance within the proteome, we calculated the top five principal components (PCs) of the data for each brain region and correlated the PCs with a line to assess genetic background effects, and AD genotype to assess transgene effects, and sex (Figures 2A, B). AD genotype was strongly correlated with the first PC in both brain regions. More variance was explained by AD genotype in the hippocampus (23%) than the cortex (16%). Line significantly correlated with more than one PC in each brain region, indicating that genetic background had broad effects on protein variance across the proteome. Variance partition analysis verified line and AD genotype as the largest drivers of variation in protein abundance in each brain region (Figures 2C, D, Supplementary Tables 3, 4). To identify proteins with the highest total variance attributable to each trait, variance partition results were sorted and ranked by protein for both the cortex and hippocampus (Supplementary Figures 1A, B). Many proteins within the top ten most variable proteins by trait overlapped between brain regions. Among the proteins with the highest attributable variance to line, proteins with significantly higher or lower abundance in B6xD2 compared to B6 could be observed, as expected (Supplementary Figure 1C). Conversely, those proteins with the highest attributable variance to AD genotype were not significantly different between genetic backgrounds (Supplemenatary Figure 1D). These results suggested that genetic background variation had a large effect on the mouse brain proteome in both the hippocampus and cortex. As illustrated in Figures 2C, D, in the cortex, the effect was comparable to that observed with 5XFAD transgene expression, whereas in the hippocampus, the effect of AD genotype on protein expression variance was greater than the effect of genetic background.

### Protein abundances driven by AD genotype and genetic background

**Figure 3:**
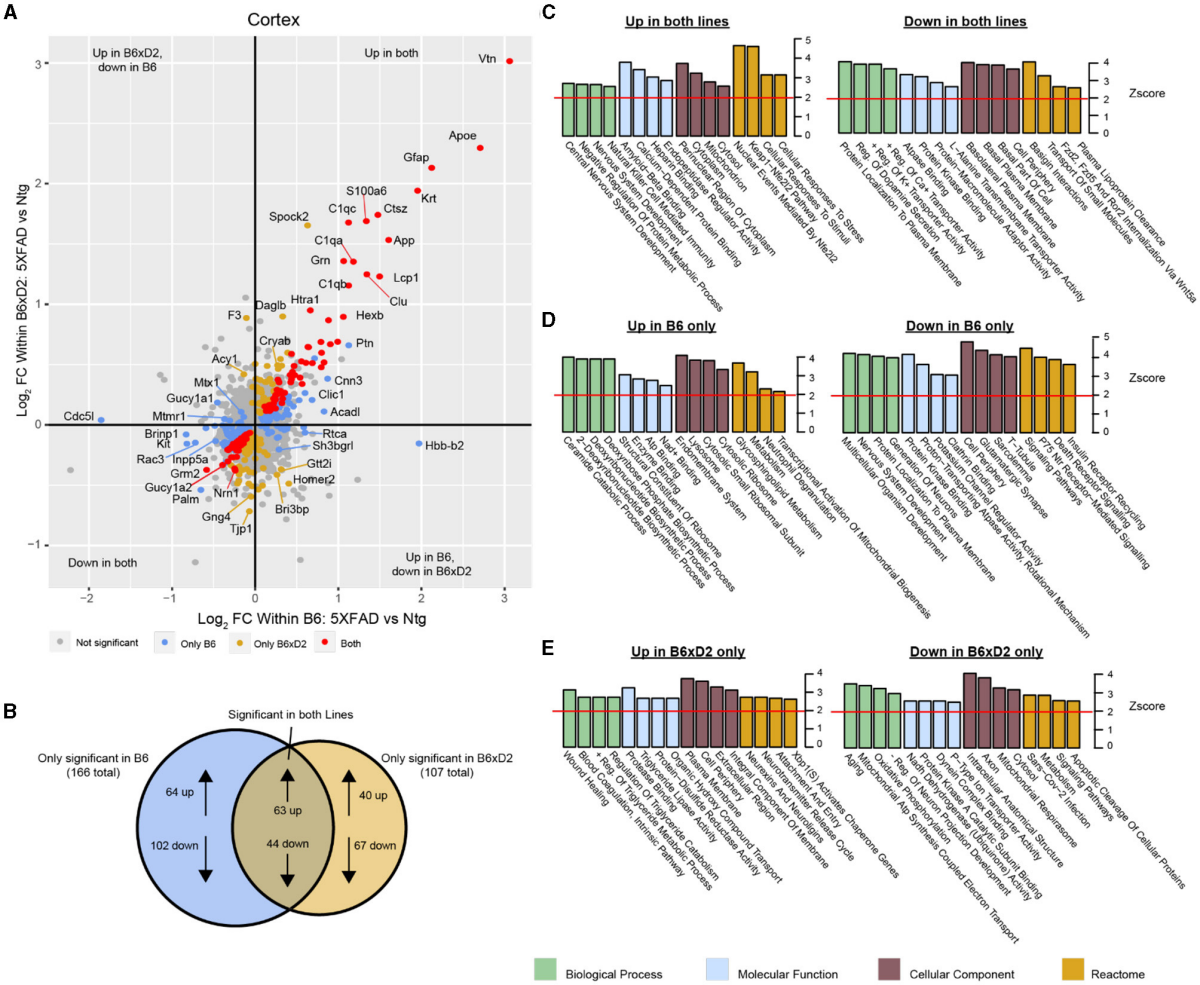
Differential protein abundance driven by genetic background and AD genotype. **(A)** Comparison of differential protein abundance between 5XFAD and Ntg mice on either the B6 or B6xD2 genetic background in the cortex. One-way ANOVA with Tukey's *post-hoc* correction was used to generate *p*-values (significance was determined as *p* < 0.05). Log_2_ fold change (FC) differences were plotted to incorporate the directionality of change in each genetic background. Color scheme: gray, not significantly changed; red, significantly changed in both B6 and B6xD2; gold, significantly changed only in B6xD2; blue, significantly changed only in B6. **(B)** Venn diagram representing the overlap of significantly differentially abundant proteins comparing 5XFAD with Ntg on either the B6 or B6xD2 genetic backgrounds (significance was determined as *p* < 0.05). **(C–E)** Gene ontology analysis of protein subsets significantly increased or decreased on both lines **(C)** or on only B6 **(D)** or only B6xD2 **(E)** lines. Ontology analysis was performed for biological processes, molecular functions, cellular components, and the reactome. A *z* score of 1.96 or higher is considered significant (red line, *p* < 0.05).

To examine how protein abundances were impacted by either AD genotype or genetic background, we assessed differential protein abundance in each brain region. Protein abundance fold changes and statistical significance between groups were calculated for all proteins in the dataset. We compared abundance changes driven by 5XFAD transgene expression in B6 and B6xD2 backgrounds in both direction and significance of change (Figure 3A). A comparable number of differentially abundant proteins was observed for both B6 and B6xD2 backgrounds when comparing 5XFAD to Ntg groups in both the cortex and hippocampus, demonstrating a similar overall effect of the transgene between mouse lines (Supplementary Tables 5, 6). The overlap of AD genotype-driven protein changes in the cortex between mouse lines was also determined (Figure 3B, Supplementary Figure 2, Supplementary Tables 7, 8). We identified a core group of proteins that were significantly changed in both mouse lines as well as protein subsets that were uniquely changed in only one mouse line or the other. Comparable findings were observed for both the cortical and hippocampal regions (Supplementary Tables 7–10). To understand the potential biological relevance of the changed proteins in each subset, gene ontology (GO) was performed in each brain region with attention to the direction of change (Figures 3C–E). Notably, “Amyloid-β binding” was among the GO terms with the highest enrichment scores from proteins increased in both backgrounds in the cortex, indicating the consistency of core AD genotype-driven protein changes regardless of genetic background. The influence of genetic variation, however, also had clear impacts on disease-relevant biological pathways. For example, the reactome term “Metabolism” was enriched in the opposite direction for each line, with B6 showing an increase and B6xD2 showing a decrease in proteins corresponding with this term. This is consistent with a previously published research in which B6 and D2 mice exhibit significantly different glucoregulatory phenotypes (Berglund et al., 2008). In addition, the directionality for some B6xD2-specific terms was unexpected in 5XFAD. For instance, the term “Aging” was decreased, and the term “Neurotransmitter release” was increased on the B6xD2 line only. In summary, we found that the proportion of changed proteins with 5XFAD transgene expression on each line appeared comparable and that key proteins affected by the expression of the transgene were not different between the B6 and B6xD2 lines. However, the impact of genetic background on AD genotype expression was significant for other proteins and pathways.

### Correlation network analysis of multi-region 5XFAD brain proteome

**Figure 4:**
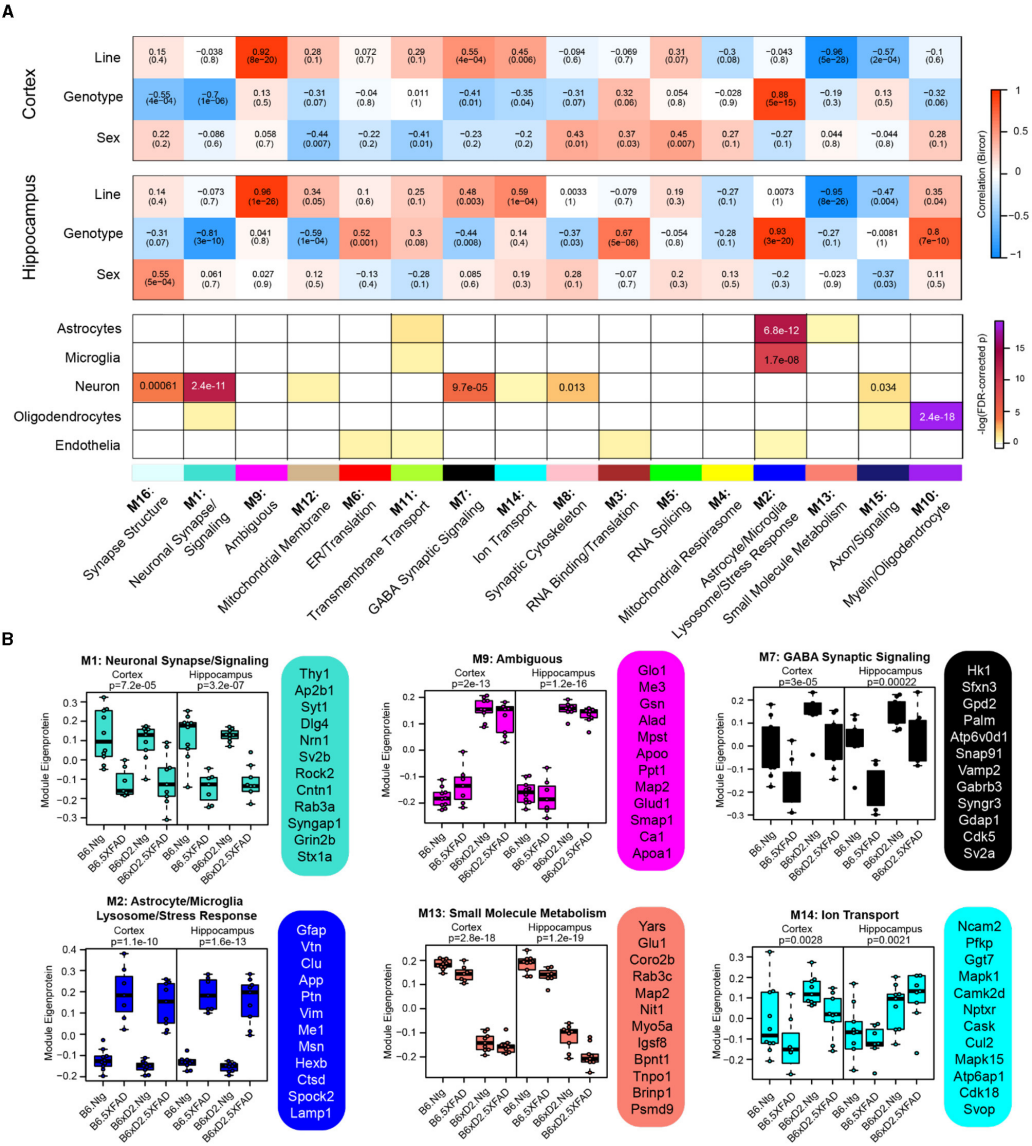
Consensus correlation network analysis of multi-region 5XFAD mouse brain proteome. **(A)** Consensus-weighted gene correlation network analysis (cWGCNA) was performed with 3,368 proteins from the cortex and hippocampus, which resulted in 16 co-expression protein modules. (Top) Correlation of module eigenproteins (MEs) with traits (line, AD genotype, and sex) for each brain region is represented by a heatmap (red, positive correlation; blue, negative correlation). (Bottom) Enrichment of cell-type markers in each module for astrocytes, microglia, neurons, oligodendrocytes, and endothelial cell types. Modules are identified by color and number as well as representative biology as determined by top gene ontology terms. **(B)** MEs for selected modules of interest grouped according to line—AD genotype pairings evaluated (B6 Ntg, B6 5XFAD, B6xD2 Ntg, and B6xD2 5XFAD) in each brain region. Hub proteins of each module are provided beside each ME box plot. MEs were compared by group in each brain region using one-way ANOVA; unadjusted *p*-values are shown. Box plots represent the median, 25th and 75th percentiles. Box hinges represent the interquartile range of the two middle quartiles in a group. Error bars are based on data points 1.5 times the interquartile range from the box hinge.

To examine biologically related groups of proteins and how they are altered by 5XFAD transgene expression and genetic background in the hippocampus and cortex, we used the consensus weighted gene correlation network analysis (cWGCNA) algorithm to build a protein co-expression network from our proteomic data (*n* = 3,368 proteins). We identified 16 clusters (modules) of highly co-expressed proteins (Figure 4A, Supplementary Tables 11, 12). Modules were very well preserved across both brain regions (Supplementary Figure 3). Representative module biology was assigned by the top GO terms for module protein members (Supplementary Table 13). Only one module (M9) was assigned as ambiguous based on the available GO terms. The approximate cell-type contribution to each module was determined by the enrichment analysis of neuronal, microglial, astrocyte, oligodendrocyte, and endothelial cell-type markers (Supplementary Tables 14, 15). There was at least one module significantly associated with each of the cell types.

Module eigenproteins (MEs) were correlated with the AD genotype, line, and sex for each brain region. MEs represent the first principal component of protein expression within each module, and thus module—trait correlations can provide information on how variables of interest are related to these protein groups (Figure 4A). We also examined ME levels across the different groups in the hippocampus and cortex (Figure 4B). Within the network, there were modules strongly driven by the primary traits line and AD genotype as well as mixed effects. Modules M1 neuronal synapse/signaling and M2 astrocyte/microglia lysosome/stress response were highly related to AD genotype, where 5XFAD transgene expression was significantly associated with decreased protein abundance in M1 members and significantly associated with increased protein abundance in M2 module members, regardless of genetic background. Notably, M1 and M2 were the largest modules in the network (M1 = 311 proteins, M2 = 246 proteins) and represented the strongest cell-type enrichments for neurons (M1), astrocytes, and microglia (M2). M9 Ambiguous and M13 small molecule metabolism were the most significantly driven by genetic background, where M9 constituents were significantly higher in B6xD2 mice compared to B6 and M13 constituents were significantly lower in B6xD2 mice compared to B6, regardless of the AD genotype. In addition, modules including M7 GABA synaptic signaling and M14 ion transport were significantly associated with more than one feature. Module M7 was significantly correlated with line and anticorrelated with AD genotype. Module M14 was correlated with line in both brain regions and anticorrelated with AD genotype only in the cortex.

In summary, large modules such as M1 neuronal synapse/signaling and M2 astrocyte/microglia lysosome/stress response indicate strong AD genotype-driven effects related to disease unimpacted by genetic background. However, multiple modules strongly driven by line were also present. Together, these findings suggest that genetic background may have more subtle or additive effects on the proteomic changes surrounding amyloidosis observed in the 5XFAD model. Additional studies are necessary to understand how modules relating to genetic background may contribute to or impact the overall AD phenotype in these mice.

### Comparison of 5XFAD and human AD brain proteomic networks

**Figure 5:**
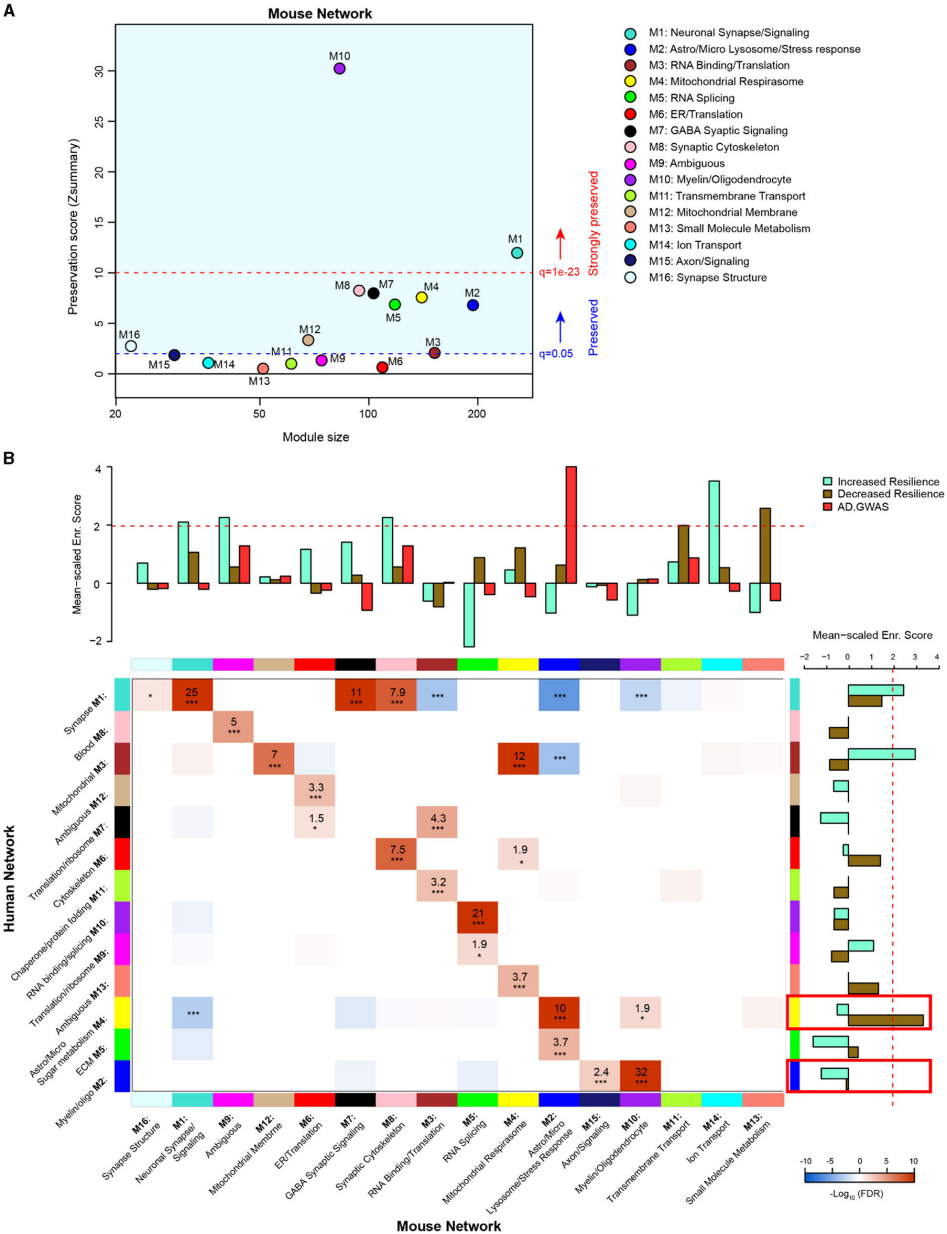
Comparison of 5XFAD mouse and human AD brain proteomic networks. **(A)** Network preservation of mouse protein network modules in a human AD frontal cortex network as published in Johnson et al. (2020). Modules with Zsummary ≥1.96 (*q* = 0.05, dashed blue line) are considered preserved, and modules with Zsummary of 10 or higher (*q* = 1e−23, dashed red line) are considered highly preserved. The majority (10 out of 16) of consensus modules from the mouse proteome were found to be preserved in the human network. **(B)** Heatmap for the overrepresentation analysis (ORA) of mouse consensus module members with human frontal cortex module members. Red indicates overrepresentation, and blue indicates underrepresentation. Numbers in boxes are –log_10_ FDR values. *P* ^*^ <0.05, ^**^ <0.01, ^***^ <0.001. Heatmap threshold is set at 10% FDR (0.1). Bar plots in the heatmap margins show the enrichment of proteins identified from GWAS of AD risk (red) or PWAS of cognitive resilience for both increased resilience (teal) or decreased resilience (olive) for mouse and human network module members. Dashed red line (*z* score 1.96 or FDR *q* ≤ 0.05) indicates the significance cutoff. Red boxes in the human enrichment bar plot indicate previously identified significantly enriched modules for AD GWAS proteins.

In order to evaluate the extent to which genetic background influences model overlap with the human disease, we compared our AD mouse model network with a human AD frontal cortex brain proteome network (Johnson et al., 2020). We used two similar but distinct analyses to compare the mouse network with the human network: (1) module preservation, which assesses the presence or absence of mouse modules in the human network based on measures of network connectivity and (2) overrepresentation analysis (ORA), which assesses the enrichment of overlapping proteins across network modules. A module preservation analysis found that the majority of modules (10/16) in the mouse network were preserved with the human network and two of these (M10 oligodendrocyte/myelin and M1 neuronal synapse/signaling) were very strongly preserved (Figure 5A). Both M10 and M1 were strongly correlated with AD genotype in both regions and, to a lesser extent, with line in the hippocampus (Figure 4A). Modules M6 ER/translation, M9 ambiguous, M11 transmembrane transport, M12 mitochondrial membrane, M13 small molecule metabolism, and M14 ion transport were not preserved with the human network modules based on network connectivity. Of the mouse modules that did not preserve with human modules, M9, M13, and M14 in particular were strongly associated with genetic background. Overrepresentation analysis found greater overlap with human modules and identified modules in the mouse network that were closely related to key human AD biology (Figure 5B, Supplementary Table 16). All but three of the mouse network modules significantly overlapped with the human modules. Modules M11 transmembrane transport, M14 ion transport, and M13 small molecule metabolism did not show significant overlap with any of the human modules, consistent with the module preservation analysis. Because our mouse cWGCNA network was a consensus network of hippocampal and cortex tissues, we also tested whether a cortex-only mouse network would show better preservation and overlap with the human cortex-only network (Supplementary Table 17). We found that 11/20 cortex-only modules were preserved in the human network, and 11/20 modules showed significant overlap by ORA, similar to the cWGCNA network findings (Supplemenatary Figure 4, Supplementary Table 18), indicating that the cross-species analyses were not significantly influenced by the inclusion of hippocampal tissue in the cWGCNA analyses. The collective results of these analyses indicate that the majority of protein co-expression relationships are preserved between mice and humans, but they also suggest there may be significant effects driven by line that are either species-specific or not well represented in this human network due to differences in proteome coverage.

To assess how the mouse network modules related to human AD genetic risk, an enrichment analysis was performed using the results from AD genome-wide association studies (GWASs) (Lambert et al., 2013; Kunkle et al., 2019; Bellenguez et al., 2022). This enrichment strategy previously identified human modules M2 myelin/oligodendrocytes and M4 astrocyte/microglia sugar metabolism as enriched with AD risk factor proteins (Johnson et al., 2020). The same analysis was performed on mouse modules, which identified M2 astrocyte/microglia lysosome/stress response as enriched with AD risk factor proteins (Supplementary Table 19). Importantly, the ORA results indicated mouse module M2 astrocyte/microglia lysosome/stress response significantly overlapped with human module M4 astrocyte/microglia sugar metabolism. This finding highlights the consistency of human disease features represented in the mouse model. Furthermore, mouse module M2 was driven strongly by AD genotype but not genetic background.

In addition to disease risk, we also used results from a proteome-wide association study (PWAS) of cognitive resilience to identify modules in both networks enriched with proteins shown to relate to cognitive performance over time (Yu et al., 2020). PWAS results were generated from dorsolateral prefrontal cortex brain tissue samples analyzed via tandem mass tag (TMT)–MS, which were adjusted for AD pathologies before determining the association between cortical protein abundance and cognitive change over time. Proteins with increased abundance and a relationship to slower rates of cognitive decline were considered to confer increased resilience, whereas proteins with higher abundance and associated with faster rates of decline were considered to confer decreased resilience. Multiple modules were identified in both human and mouse networks for increased and decreased resilience proteins. There were four mouse modules enriched for proteins associated with increased cognitive resilience: M1 neuronal synapse/signaling, M9 ambiguous, M8 synaptic cytoskeleton, and M14 ion transport (Supplementary Table 20). There were also two mouse modules enriched for proteins associated with decreased resilience: M11 transmembrane transport and M13 small molecule metabolism (Supplementary Table 21). In the human network, there were two modules enriched for increased resilience proteins: M1 synapse and M3 mitochondrial (Supplementary Table 22). Only one module was enriched in the human network for decreased resilience proteins: M4 astrocyte/microglia sugar metabolism (Supplementary Table 23).

Finally, because our network did not provide information on causality beyond that suggested by AD GWAS overlap, we integrated our mouse network with a directional Bayesian network of the human brain to infer potential causal changes in the mouse network related to AD (Ding et al., 2021). We found 88 proteins in the cWGCNA mouse network that overlapped with key driver proteins in the Bayesian human brain network (Supplementary Table 24). Interestingly, most of the overlapping proteins mapped to the M1 neuronal synapse/signaling module, followed by the M10 myelin/oligodendrocyte module. As noted above, M1 neuronal synapse/signaling was significantly decreased by 5XFAD transgene expression and was enriched in proteins that are associated with resilience to AD. M1 also highly overlaps with the human M1 synapse module. Therefore, the disruption of M1 neuronal synapse/signaling by the 5XFAD transgene may lead to many downstream brain proteome changes observed in this model. Given the strong overlap between mouse and human M1 modules, the disruption of M1 in humans by mechanisms potentially shared between 5XFAD transgene manipulation and human AD may also cause many of the widespread proteome changes observed in the human AD brain.

In summary, consistency in enrichment results could be observed between networks; however, there were also distinct, non-overlapping features. Mouse module M2 astrocyte/microglia lysosome/stress response, which was enriched for AD GWAS markers, most strongly overlapped with human module M4 astrocyte/microglia sugar metabolism, which was the top GWAS module in the human network (Johnson et al., 2020). Mouse modules M1 neuronal synapse/signaling, M9 ambiguous, and M8 synaptic cytoskeleton were all enriched with increased resilience markers and strongly overlapped with human module M1 synapse, which was consistently enriched with increased resilience markers. Notably, mouse modules M13 small molecule metabolism, M11 transmembrane transport, and M14 ion transport were all significantly enriched for either increased or decreased resilience protein markers; however, these modules had no significant overlap with human modules. Overlap with a Bayesian human brain network suggested that M1 neuronal synapse/signaling disruption may drive many downstream proteomic network changes in AD.

## Discussion

In this study, we examined the brain proteomes of mice from two genetic backgrounds across two brain regions of 5XFAD and Ntg animals to assess the impact of incorporating genetic diversity on the proteome in mouse models of AD. We found that both genetic background and transgene expression strongly contributed to variance in the proteome. The strongest protein changes driven by the 5XFAD transgene were not significantly altered by genetic background at either the single protein or protein network levels. However, genetic background did influence proteins relevant to disease related to synaptic biology and mitochondrial processes, among others. Together, these results indicate that, as expected, the classical disease-relevant changes related to 5XFAD transgene expression are robust; however, proteins significantly correlated with genetic background may represent individualized targets related to novel genetic factors and disease subtypes, and may also provide insights into resilient and susceptible phenotypes. Network analysis also facilitated the comparison of mouse and human co-expression brain proteomes, in which the majority of the mouse modules were preserved and overlapped with human network modules. Similar to the 5XFAD transgene findings, mouse proteome modules that overlapped most strongly with human disease were largely unaffected by genetic background. Overall, this study contributes to ongoing efforts to rigorously validate and characterize AD model systems using multiple different–omics approaches and illustrates the potential effects on the mouse proteome of introducing genetic variability for improving cross-species translation.

Previous studies have documented line-dependent changes in pathological and phenotypic measures, including Aβ deposition and learning and memory tasks (Ryman et al., 2008; Sultana et al., 2019). Consistently, the brain proteome comparing 5XFAD with Ntg animals demonstrated line-specific differences at the individual protein and co-expression levels. Among these line-specific differences, biology relating to synaptic function could suggest potentially important changes introduced by genetic variability relevant to human neurodegenerative conditions. Specifically, the ontology terms “Neurotransmitter release cycle” and “Neurexins and neuroligins” were significantly changed in B6xD2 animals. In addition, module M7 GABA synaptic signaling was strongly correlated with lines. Changes in synaptic integrity and density are believed to underlie cognitive symptoms in human disease, and more recently, the ability to maintain functional synaptic connections has been proposed as a mechanism for supporting cognitive resilience in AD (Walker and Herskowitz, 2020; Hurst et al., 2023). Inherent differences in synaptic biology introduced by genetic variability in mouse models of AD could represent a novel opportunity to investigate human-relevant alterations in neuronal communication. The extent to which genetic diversity affects synaptic changes requires further study.

One important aspect of this study is the inclusion of more than one brain region for analysis. We found that the hippocampus was more strongly affected by transgene expression than the cortex. Principal component and variance partition analyses demonstrated that both AD genotype and line strongly contribute to variance in the proteome. However, the strength of this association was muted when the two brain regions were not separated, in which region became the strongest explanation of variance in the model (data not shown). Consensus network analysis also identified multiple modules strongly influenced by the brain region, including module M10 myelin/oligodendrocyte, in which correlation with the AD genotype was inversed by region. Collectively, these findings indicate the strength of evaluating multiple brain regions when validating specific models.

Previous methods of introducing genetic complexity in AD mouse models include the use of wild-derived lines (Onos et al., 2019). Genetically diverse, wild-derived transgenic mice produced strong differentiation of phenotypes associated with important human disease features, including cognitive-like changes, neurodegeneration, and amyloid dynamics. However, this method of introducing genetic diversity has been cautioned due to the lack of rigorous characterization in the absence of AD transgenes (Ammassari-Teule, 2022). The use of fully inbred parental lines, such as B6 and D2, which have been extensively characterized, overcomes this ambiguity. Therefore, the genetic variability introduced by crossing the B6 and D2 lines would allow the differentiation of highly heritable phenotypes and traits relating to risk and resilience in AD progression (Neuner et al., 2019). Moreover, this strategy is not limited specifically to the B6 and D2 lines but rather provides an adaptable reference for the utility of this strategy for future model development and characterization. Taken together, introducing genetic complexity by crossing widely used inbred mice represents a potentially advantageous research tool for both improving the recapitulation of complex human phenotypes and practical reproducibility.

Comparing the mouse and human networks highlighted important findings: (1) mouse modules most strongly representative of human disease were driven by 5XFAD transgene overexpression and not significantly influenced by genetic background and (2) certain mouse modules did not overlap with the human network at all (M11 transmembrane transport, M14 ion transport, and M13 small molecule metabolism). Mouse module M2 astrocyte/microglia lysosome/stress response was enriched for AD GWAS markers and corresponded most significantly to human module M4 astrocyte/microglia sugar metabolism, which was also enriched for AD GWAS markers. This overlap of disease-relevant modules enriched for glial cells is well supported in the literature (Maria et al., 2022). Similarly, mouse module M1 neuronal synapse/signaling overlapped significantly with human module M1 synapse, both of which were enriched for markers of increased cognitive resilience. M1 neuronal synapse/signaling was enriched for key drivers of brain protein expression and decreased by 5XFAD transgene expression. These mouse modules (M2 astrocyte/microglia lysosome/stress response and M1 neuronal synapse/signaling) were significantly correlated with AD genotype, but not genetic background. These findings suggest that in this model, there are core pathologies and features at the proteomic level related to human disease that are strongly driven by 5XFAD transgene expression, as expected. As AD genetic risk clusters in the M2 mouse and the corresponding M4 human module, dysfunction in these modules may subsequently affect M1 mouse and human modules enriched in key drivers of brain protein expression, leading to further downstream pathology. Although these modules were not affected by genetic background, other modules that are strongly driven by genetic background could provide putative targets for understanding individual differences driven by genetic variants that may inform mechanisms of resilience or susceptibility. In addition, the lack of overlap between specific mouse modules (M11 transmembrane transport, M14 ion transport, and M13 small molecule metabolism) might also be explained by the current transgenic model utilized. Another consideration for this gap is the comparative depth of LFQ vs. TMT proteomics. Both mouse and human networks compared here were analyzed via LFQ proteomics; however, the PWAS results integrated into the enrichment analysis were generated using TMT-MS. This offers a potential explanation for the significant enrichment of M11, M14, and M13 with proteins associated with cognitive trajectory despite their lack of overlap with human modules. Future proteomic studies on APP knock-in models using TMT-MS to increase proteome depth of coverage may allow for more sensitive detection of genetic background effects on disease-relevant proteomic changes.

Some potential limitations of our study should be noted. First, the current cohort of animals was aged 12–14 months, a timepoint in which significant amyloid pathology has developed in the 5XFAD model. While evaluating genetic contributions is valuable at this stage in the lifespan, measuring proteomic differences at one static timepoint may miss transient or time-dependent pathological changes. Future studies evaluating multiple timepoints could, therefore, provide valuable insights into the rates of proteomic changes that may be influenced by mixed genetic backgrounds. Second, one of the goals of introducing genetic complexity in mouse models is to generate phenotypically distinct substrains that enable the characterization of novel genetic variants contributing to cognitive impairment. In this study, we analyzed only one mixed genetic background (B6xD2), and behavioral data were not included in the analysis. Analysis of larger cohorts of up to 50 AD-BXD lines that better model the heterogeneity of humans is underway and will provide sufficient power to interpret the mediation of the proteome on brain and cognitive phenotypes (Neuner et al., 2019; Johnson et al., 2023). These rich data are being generated and shared from consortium initiatives, such as Resilience-AD and Model Organism Development and Evaluation for Late-Onset Alzheimer's Disease (MODEL-AD) (Oblak et al., 2021).

Mice represent an ideal model system due to their combination of phylogenetic conservation with humans and their relative ease in experimental manipulation and management. Currently, there are approximately 200 (and counting) transgenic models of AD that have been developed, the majority of which utilize FAD mutations expressed on the fully inbred B6 genetic background. This schema for model development has led to invaluable insights into the core mechanisms of AD progression and risk, but there remains a need for improvement in the translational validity of AD mouse models. Proteomics provides one level of analysis into a complex, polygenic human disease. Incorporation and careful consideration of other levels of analysis, such as the transcriptome (Maria et al., 2022), can provide additional insights into the effect of genetic variability on AD phenotypes for the advancement of both model translatability and therapeutic target nomination. Our results suggest that 5XFAD transgene expression induces robust changes in the mouse brain proteome, but that the addition of genetic background complexity introduces significant proteomic variance that may contribute to phenotypic variability and alignment with the human disease. Therefore, this approach remains a promising avenue to improve face validity in other AD mouse models, such as knock-in models, where proteomic changes may be more subtle than in transgenic models.

### Data and code availability

Raw mass spectrometry data and database search results from cortex and hippocampus mouse brain tissue analysis can be found at: https://www.synapse.org/B6xD2proteomics Processed data and code are also provided. Human MS data were previously shared and can be accessed at: https://www.synapse.org/consensus and https://www.synapse.org/DeepConsensus.

## Methods and materials

### Mice

Female hemizygous 5XFAD mice on a congenic C57BL/6J background (RRID: MMRC_034848-JAX) were bred to male C57BL/6J (RRID: IMSR_JAX:000664) or DBA/2J mice (with corrected *Gpnmb* mutation, RRID: IMSR_JAX:007048). Animals were kept on a 12:12 light:dark cycle and were provided food and water *ad libitum*. All routine procedures were approved by the Institutional Care and Use Committee (IACUC) at The Jackson Laboratory and in accordance with the standards of the Association for the Assessment and Accreditation of Laboratory Animal Care (AAALAC) and the National Institutes of Health Guide to the Care and Use of Laboratory Animals. Mice (*n* = 10 B6 5XFAD, *n* = 10 B6xD2 5XFAD, *n* = 10 B6 Ntg, *n* = 10 B6xD2 Ntg; five males, five females per group) at 14 months of age (±2 wks) were deeply anesthetized with isoflurane, rapidly decapitated, and the brain removed. The hippocampus and frontal cortices were isolated on an ice-cold dissecting block and immediately frozen in liquid nitrogen.

### Tissue homogenization

Tissue dissections of the cortex and hippocampus were each homogenized in urea lysis buffer (8 M urea, 100 mM NaH2PO4, pH 8.5) at a 1:5 weight-to-buffer ratio with HALT phosphatase and protease inhibitor cocktail (1X final concentration, Pierce). Samples were homogenized in RINO sample tubes (Next Advance) with ~100 mL of stainless-steel beads using a Bullet Blender (Next Advance) at 4° for two full 5-min cycles. The homogenates were transferred to clean, Eppendorf LoBind tubes and sonicated for three cycles at 5 s on and 5 s off at 20% amplitude on ice. The samples were then centrifuged for 5 min at 15,000 × *g*, and the supernatant was transferred to clean tubes. Protein concentrations were determined using the bicinchoninic acid assay (BCA, Pierce). One-dimensional SDS-PAGE gels were run, followed by Coomassie blue staining as quality control to ensure protein integrity and equal loading.

### SDS-PAGE

A total of 20 mg of protein from each sample was mixed with Laemmli sample buffer (Bio-Rad) and β-mercaptoethanol before being boiled at 95° for 10 min, spun briefly to collect volume, and loaded into Bolt 4–12% Bis-Tris gradient gels (Invitrogen). Loaded gels were initially electrophoresed at 80 mV for the lowest percentage gradient of the gel, followed by 120 mV for the remainder of the gel. The gels were then submerged in Coomassie blue staining overnight and destained briefly the following day to visualize protein banding (Supplemenatary Figure 5).

### Western blotting

Two female mouse samples were selected per experimental group. Cortical mouse brain lysate was normalized to 20 μg in 12 mL of a final concentration of 1X Laemmli sample buffer (Bio-Rad) and 355 mM β-mercaptoethanol (Sigma-Aldrich). The samples were boiled at 95°C for 10 min prior to loading into 1.0 mm 4–12% Bis-Tris NuPAGE 10-well gels (Invitrogen) with a broad range of 10–250 kDa molecular weight markers (New England Biosciences, P7719). The gel was run at 80 V for 15 min, then increased to 125 V until the molecular weight marker reached the end of the gel. Following gel electrophoresis, proteins were transferred to a 0.2-mm nitrocellulose membrane at 20 V for 7 min and prepared in accordance with the iBlot dry transfer stack system. The membrane was incubated with 0.5% Ponceau S stain to verify equal protein loading. The membrane was then washed twice with Tris-buffered saline (TBS) to de-stain. The membrane was blocked for 30 min in StartingBlock Blocking Buffer (Thermo Fisher Scientific) to reduce non-specific binding. The membrane was incubated on an orbital shaker overnight at 4°C with primary antibodies. The following primary antibodies were used for validation: Dako polyclonal rabbit anti-GFAP (Cat #Z0334) and Chemicon polyclonal goat anti-ApoE (Cat #AB947). Abcam rat monoclonal [YL1/2] (Cat #ab6160) was used as a loading control. All antibodies were diluted 1:1000 in StartingBlock buffer prior to incubation. After overnight incubation, the membrane was washed twice for 10 min with TBS-T to remove unbound primary antibodies. Invitrogen Donkey anti-Rat Alexa Fluor 488, Donkey anti-goat Alexa Fluor 680, and Donkey anti-rabbit Alexa Fluor 800 conjugated secondary antibodies were used to detect and visualize proteins of interest. All secondary antibodies were diluted 1:10,000 in a StartingBlock buffer prior to incubation and incubated with the membrane on an orbital shaker for 1 h at room temperature. The membrane was washed with TBS-T twice for 10 min to remove unbound antibodies and twice for 5 min in TBS before acquiring images on the Licor Odyssey M system. Relative protein band intensities were quantified using ImageJ FIJI software. Membrane images were uploaded in FIJI, and band intensities were obtained by “Mean Gray Value” measurements. The entire protein band per lane was selected as the region of interest (ROI). The same ROI selection area was used for the quantification of bands between lanes of the same protein. ROIs above or below the band of interest were used to obtain background intensity values. Pixel density intensities were inverted by subtracting the band value from 255, and the net protein value was obtained by subtracting the inverted background value from the inverted band value. The signal for each protein was then divided by the α-tubulin signal for its respective lane for loading normalization. Results of Western blotting for APOE and GFAP are provided in Supplementary Figure 2.

### Protein digestion

A total of 100 mg of protein from each sample was aliquoted and volume normalized to 50 mL in a urea lysis buffer before being reduced with 5 mM dithiothreitol (DTT) for 30 min at room temperature, followed by alkylation with 10 mM iodoacetamide (IAA) for 30 min under light protection. The samples were digested overnight with 1:50 (w/w) lysyl endopeptidase (Wako). The following day, urea concentration for each sample was diluted to < 1 M using ammonium bicarbonate (ABC) and digested for an additional ~16 h using 1:50 (w/w) trypsin (Promega). Following digestion, peptides were acidified to a final concentration of 1% (v/v) formic acid (FA) and 0.1% trifluoroacetic acid (TFA) and desalted using 30 mg HLB columns (Oasis). Prior to sample loading, each HLB column was rinsed with 1 mL of methanol, washed with 1 mL of 50% (v/v) acetonitrile (ACN), and equilibrated (x2) with 1 mL of 0.1% (v/v) TFA. The samples were then loaded onto the columns and washed (x2) with 1 mL of 0.1% (v/v) TFA. The peptides were eluted with two volumes of 0.5 mL of 50% (v/v) ACN. Eluates were frozen at −80°C overnight before being completely dried using a SpeedVac (Labconco).

### LC-MS/MS

All samples (~1 mg) were loaded and eluted using an Ultimate RSLCnano (Thermo Fisher Scientific) with an in-house packed 15 cm, 150 mm i.d. capillary column with 1.7 mm C18 CSH (Waters) over a 45 min gradient. The gradient went from 1 to 99% Buffer B (Buffer A: water in 0.1% formic acid and Buffer B: 80% acetonitrile in 0.1% formic acid). Mass spectrometry was performed with an Orbitrap Lumos (Thermo) in positive ion mode using data-dependent acquisition with 3 s top speed cycles. Each cycle consisted of one full MS scan followed by as many MS/MS events as could fit within the given 1 s cycle time limit. MS scans were collected at a resolution of 120,000 (375–1,500 m/z range, 4 × 10^5^ AGC, 50 ms maximum ion injection time). Only precursors with charge states between 2+ and 6+ were selected for MS/MS. All higher energy collision-induced dissociation (HCD) MS/MS spectra were acquired at a resolution of 15,000 (1.6 m/z isolation width, 35% collision energy, 1 × 10^5^ AGC target, 22 ms maximum ion time). Dynamic exclusion was set to exclude previously sequenced peaks for 30 s within a 10-ppm isolation window.

### Database searching and protein quantification

Quantitation was performed as previously published (Seyfried et al., 2017), with a slight modification: RAW data files from all 80 samples were analyzed using MaxQuant (v1.6.17.0) using a mouse database (91,415 target sequences, downloaded August 2020). Methionine oxidation, asparagine and glutamine deamidation, protein N-terminal acetylation, and serine, threonine, and tyrosine phosphorylation were variable modifications (up to five allowed per peptide); cysteine was assigned as a fixed carbamidomethyl modification (+57.0215 Da). A precursor mass tolerance of ±20 ppm was applied prior to mass accuracy calibration and ±4.5 ppm after internal MaxQuant calibration. Other search settings included a maximum peptide mass of 6,000 Da, a minimum peptide length of six residues, and a 0.05 Da tolerance for high-resolution MS/MS scans. The false discovery rate (FDR) for peptide spectral matches, proteins, and site decoy fractions was all set to 1%. Quantification settings were as follows: re-quantification with a second peak finding attempt after protein identification has been completed; match full MS1 peaks between runs; and a 0.7-min retention time match window should be used after an alignment function was found with a 20-min retention time search space. Only razor and unique peptides were considered for protein-level quantitation as summed intensities.

### Data preprocessing

A total of 4,351 high-confidence master proteins were identified and quantified across all 80 samples. Protein abundances were log2 transformed before sample outlier detection and missingness filtering. Network connectivity identified five samples as outliers [samples >3 standard deviations away from the mean, as previously described (Johnson et al., 2020, 2022)] that were removed along with the matched tissue pairs from the other brain region, and only proteins with >50% missing values across samples were included. The final data matrix included 70 samples and 3,368 proteins for downstream analyses.

### Differential expression analysis

Significantly differentially changed proteins between groups were defined using one-way ANOVA with Tukey's comparison *post-hoc* test (significance was determined as *p* < 0.05). Differential expressions displayed as volcano plots were generated using the ggplot2 package (3.3.5).

### Consensus weighted gene correlation network analysis (cWGCNA)

Network analysis was performed using the consensus configuration of the weighted gene correlation network analysis (cWGCNA, version 1.69) algorithm to identify co-expression modules present and shared in both cortex and hippocampal brain regions. The WGCNA::blockwiseConsensusModules function was run with soft threshold power at 7.0, a deep split of 4, a minimum module size of 30, merge cut height at 0.07, a mean topological overlap matrix (TOM) denominator, bicor correlation, signed network type, pamStage, and pamRespectsDendro parameters both set to TRUE and a reassignment threshold of 0.05. This function calculates pairwise biweight mid-correlations (bicor) between protein pairs. The resulting correlation matrix is then transformed into a signed adjacency matrix, which is used to calculate a topological overlap matrix (TOM), representing expression similarity across samples for all proteins in the network. This approach uses hierarchical clustering analysis and dynamic tree cutting to identify co-expression modules. Following construction, module eigenprotein (ME) values were defined. The MEs are the first principle component of a given module and are considered representative abundance values for a module that also explains modular protein covariance (Langfelder and Horvath, 2008). Pearson's correlation between proteins and MEs was used as a module membership measure, defined as kME.

### Gene ontology (GO) and cell-type marker enrichment analyses

Gene ontology (GO), WikiPathways, Reactome, and molecular signatures database (MSigDB) term enrichment in our gene sets of mouse network module members and significantly differentially changed protein subsets were determined by gene set enrichment analysis (GSEA) using an in-house developed R script (https://github.com/edammer/GOparallel). Briefly, this script performs one-tailed Fisher's exact tests (FET) enabled by functions of the R piano package for ontology enrichment analysis on gene sets downloaded from http://baderlab.org/GeneSets, which is maintained and updated monthly to pull in current gene sets from more than 10 different database sources including those mentioned above (Varemo et al., 2013; Reimand et al., 2019). Redundant core GO terms were pruned in the GOparallel function using the minimal_set function of the ontologyIndex R package (Greene et al., 2017). Ontology analyses were adjusted using the Benjamini–Hochberg procedure. Cell-type enrichment was also investigated, as previously published (McKenzie et al., 2017; Seyfried et al., 2017; Johnson et al., 2022). An in-house marker list combined with previously published cell-type marker lists from Sharma et al. (2015) and Zhang et al. (2014) was used for the cell-type marker enrichment analysis for each of the five cell types assessed (neuron, astrocyte, microglia, oligodendrocyte, and endothelial). If, after the lists from Sharma et al. and Zhang et al. were merged, a gene symbol was assigned to two cell types, we defaulted to the cell type defined by the Zhang et al. list, such that each gene symbol was affiliated with only one cell type. Fisher's exact tests were performed using the cell-type marker lists to determine cell-type enrichment and were corrected by the Benjamini–Hochberg procedure.

### Network preservation

Network preservation was determined using the WGCNA::modulePreservation() function. Zsummary composite preservation scores were calculated using the mouse network as the test network, the human network, and the reference network. Parameters included: 500 permutations, a random seed set to 1 (for reproducibility), and quickCor set to 0.

### Enrichment analysis of proteome-wide association study (PWAS) and genome-wide association study (GWAS) results

The proteome-wide association study (PWAS) of the cognitive trajectory by Yu et al. (2020) tested 8,356 proteins for correlation with the change in cognition over time. Unique gene symbols representing protein gene products positively correlated (*n* = 645) and negatively correlated (*n* = 575) with cognitive resilience were split into lists with corresponding *P*-values. For GWAS of AD risk, compiled single-nucleotide polymorphism (SNP) summary statistics were used [(Lambert et al., 2013; Kunkle et al., 2019; Bellenguez et al., 2022), MAGMA.SPA/MAGMAinput.zip at main · edammer/MAGMA.SPA · GitHub]. These lists were separately checked for enrichment in both mouse and human network modules using a permutation-based test (10,000 permutations) implemented in R, with exact *P*-values for the permutation tests calculated using the permp function of the statmod package (1.4.36). Module-specific mean *P*-values for enrichment were determined as a *Z*-score, specifically as the difference in mean *P*-value of gene product proteins hitting a module at the level of gene symbol minus the mean *P*-value of genes hit in the 10,000 random replacement permutations, divided by the standard deviation of *P*-value means also determined in the random permutations (Supplementary Tables 19–23).

### Mergeomics key driver analysis

Mergeomics v2.0 (http://mergeomics.research.idre.ucla.edu/runmergeomics.php) weighted key driver analysis (KDA) (Ding et al., 2021) was run using either hippocampus or cortex network non-gray gene product proteins (N = 1,897 in either), converted to human gene nomenclature (HUGO) official gene symbols via the biomaRt package's getLDS function in R v4.2.3. Each symbol was input with its corresponding mouse network module color. The other input for KDA was the Mergeomics v2.0 sample human Bayesian brain network consisting of over 29,000 nodes and ~140,000 edges as the substrate network. Parameters used were a search depth of 3 (maximum edge distance starting from each candidate key driver gene); edge type = directed, thereby leveraging the edge directionality of the input Bayesian network; minimum hub overlap of 0.33 (default); and an edge factor of 0.1, allowing the edge weights in the Bayesian network input to carry into the analysis with low to moderate impact. The table output of the key driver candidate subset from brain network hubs was further filtered to report the 88 nodes that were both FDR-adjusted significant key driver hub nodes and present in the input hippocampus or cortex gene lists. This output table was unchanged whether the input for KDA was the hippocampus or cortex module assignments, where only 34 of the 1,897 gene product proteins were in a different module comparing the hippocampus to cortex network non-gray module assignments.

### Additional statistical analyses

All proteomic statistical analyses were performed in R (version 4.0.3). Box plots represent the median and 25th and 75th percentile extremes; the hinges of a box represent the interquartile range of the two middle quartiles of data within a group. Error bars extents are defined by the farthest data points up to 1.5 times the interquartile range away from the box hinges. Correlations were performed using the biweight midcorrelation function from the WGCNA package.

## Data availability statement

The data presented in the study are deposited in the Synapse.org repository, accession numbers syn51475919 (https://www.synapse.org/B6xD2proteomics), syn20933797 (https://www.synapse.org/consensus), and syn25006611 (https://www.synapse.org/DeepConsensus).

## Ethics statement

The animal study was approved by Institutional Care and Use Committee (IACUC) at the Jackson Laboratory. The study was conducted in accordance with the local legislation and institutional requirements.

## Author contributions

CH, AD, CK, and EJ designed the experiments. CH, AD, SS, and DD carried out experiments. CH, ED, SS, DD, and EJ analyzed data. AD, NS, and CK provided advice on the interpretation of data and manuscript review. CH and EJ wrote the manuscript with input from co-authors. All authors contributed to the article and approved the submitted version.
