# Supplementary material for: Genetic background influences the 5XFAD Alzheimer's disease mouse model brain proteome
Source: Front Aging Neurosci. 2023 Oct 12;15:1239116. doi: 10.3389/fnagi.2023.1239116 (PMC10602695; doi:10.3389/fnagi.2023.1239116)
Supplement: Supplementary file 2 [file Image_1.pdf]

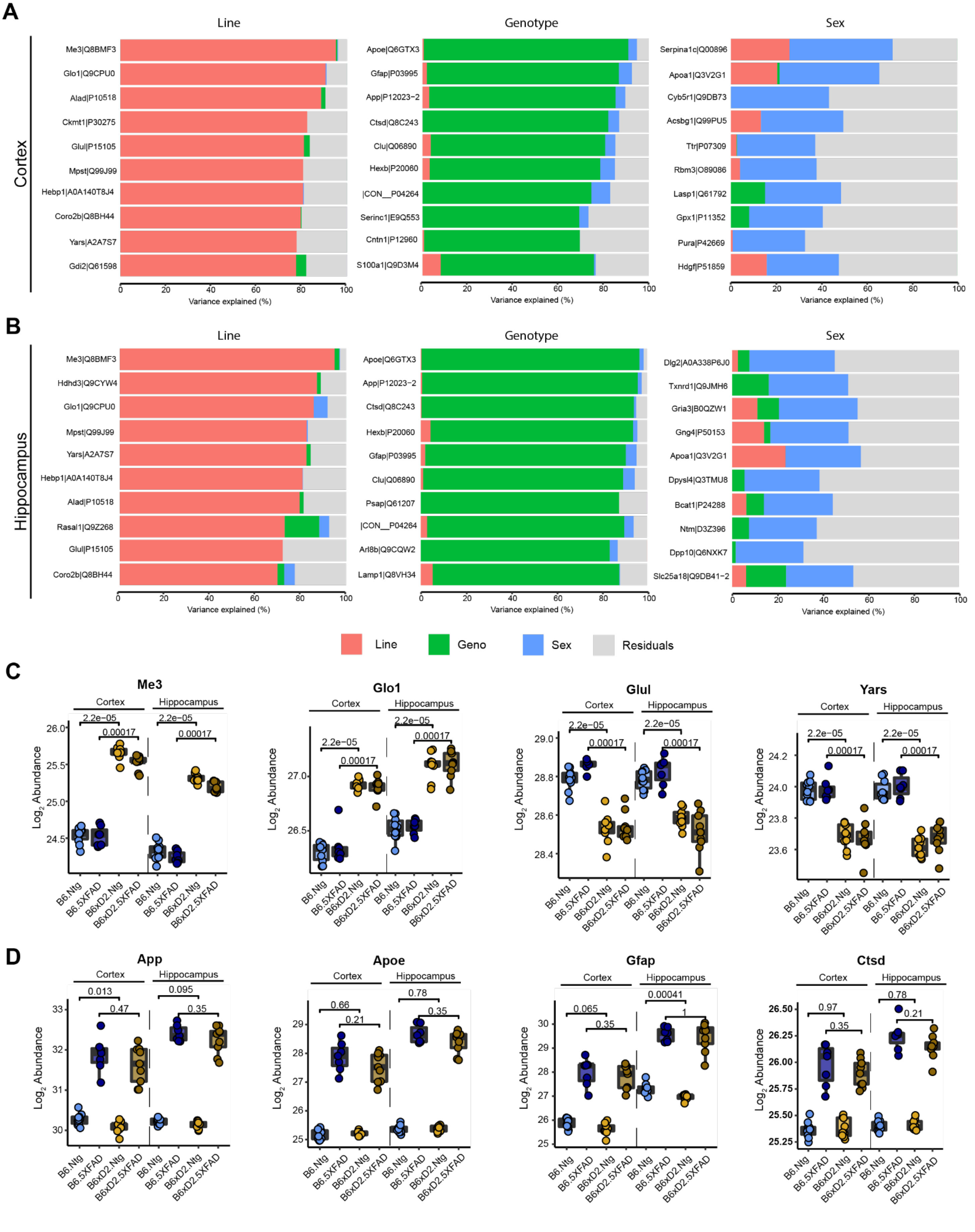
A
Line
Genotype
Sex
Cortex
Me3|Q8BMF3
Glo1|Q9CPU0
Alad|P10518
Ckmt1|P30275
Glul|P15105
Mpst|Q99J99
Hebp1|A0A140T8J4
Coro2b|Q8BH44
Yars|A2A7S7
Gdi2|Q61598
Apoe|Q6GTX3
Gfap|P03995
App|P12023−2
Ctsd|Q8C243
Clu|Q06890
Hexb|P20060
|CON__P04264
Serinc1|E9Q553
Cntn1|P12960
S100a1|Q9D3M4
Serpina1c|Q00896
Apoa1|Q3V2G1
Cyb5r1|Q9DB73
Acsbg1|Q99PU5
Ttr|P07309
Rbm3|O89086
Lasp1|Q61792
Gpx1|P11352
Pura|P42669
Hdgf|P51859
Variance explained (%)
B
Hippocampus
Me3|Q8BMF3
Hdhd3|Q9CYW4
Glo1|Q9CPU0
Mpst|Q99J99
Yars|A2A7S7
Hebp1|A0A140T8J4
Alad|P10518
Rasal1|Q9Z268
Glul|P15105
Coro2b|Q8BH44
Apoe|Q6GTX3
App|P12023−2
Ctsd|Q8C243
Hexb|P20060
Gfap|P03995
Clu|Q06890
Psap|Q61207
|CON__P04264
Arl8b|Q9CQW2
Lamp1|Q8VH34
Dlg2|A0A338P6J0
Txnrd1|Q9JMH6
Gria3|B0QZW1
Gng4|P50153
Apoa1|Q3V2G1
Dpysl4|Q3TMU8
Bcat1|P24288
Ntm|D3Z396
Dpp10|Q6NXK7
Slc25a18|Q9DB41−2
Line
Geno
Sex
Residuals
C
Me3
Glo1
Glul
Yars
Cortex
Hippocampus
Log2 Abundance
2.2e−05
0.00017
B6.Ntg
B6.5XFAD
B6xD2.Ntg
B6xD2.5XFAD
D
App
Apoe
Gfap
Ctsd
0.013
0.47
0.095
0.35
0.66
0.21
0.78
0.065
0.00041
1
0.97

Supplementary Figure 1. Stacked bar plots illustrating the top 10 proteins explaining greatest variance for line, AD genotype and sex in cortex (**A**) and hippocampus (**B**). (**C**) Top variance explained proteins for line across cortex and hippocampus. **(D**) Top variance explained proteins for AD genotype across cortex and hippocampus.

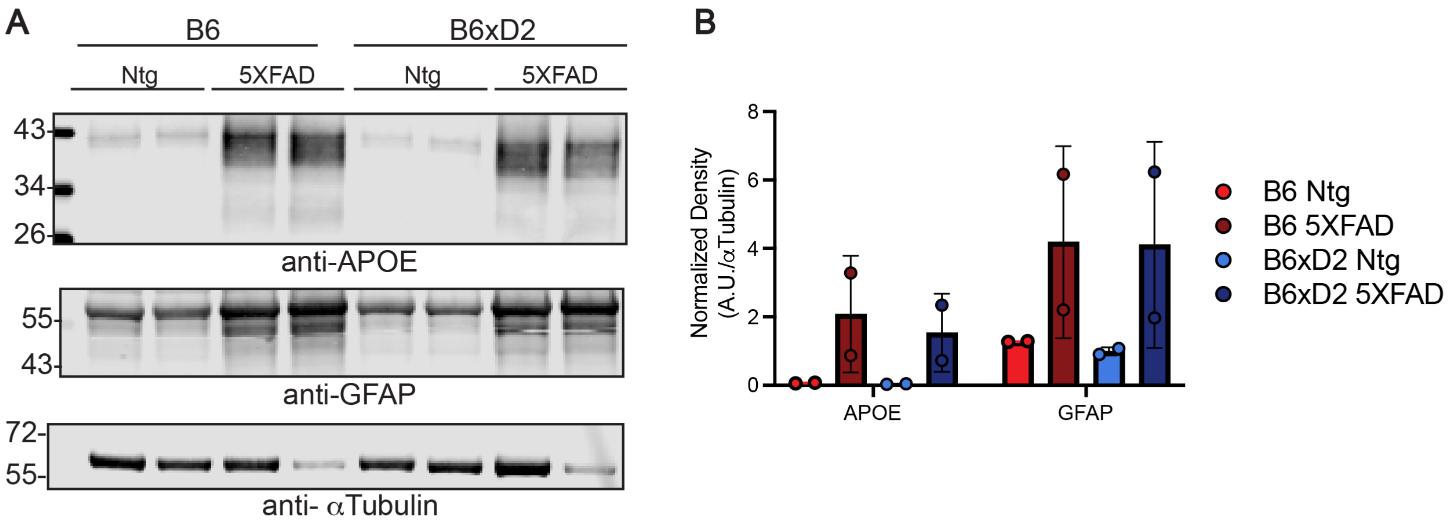

Supplementary Figure 2. (A) Levels of APOE and GFAP were assessed by Western blotting in nontransgenic (Ntg) and 5XFAD mice on the B6 and B6xD2 backgrounds in cortex. α-tubulin was used as a loading control. **(B)** Quantification of protein levels in (A).

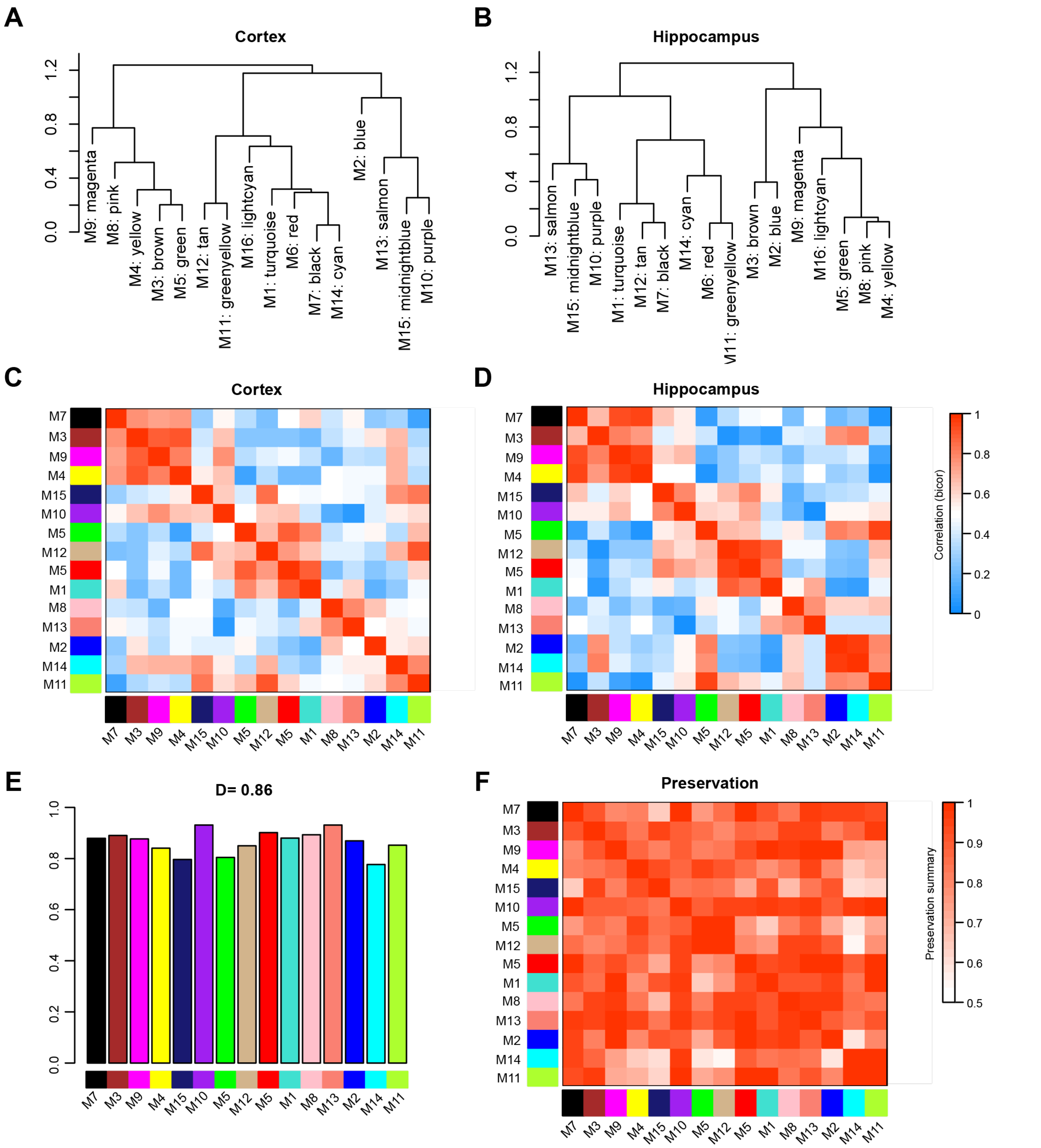

Supplementary Figure 3. (**A-B**) Consensus module eigneprotein clustering represented as a dendrogram for cortex (**A**) and hippocampus (**B**). (**C-D**) Module eigenprotein correlation heatmap for cortex (**C**) and hippocampus (**D**). (**E**) Mean preservation relationship for each eigenprotein was calculated for the consensus network. Mean preservation was 0.83, indicating a high degree of preservation. (**F**) Preservation adjacency of the consensus network represented by heatmap. Most relationships were highly preserved across brain regions.

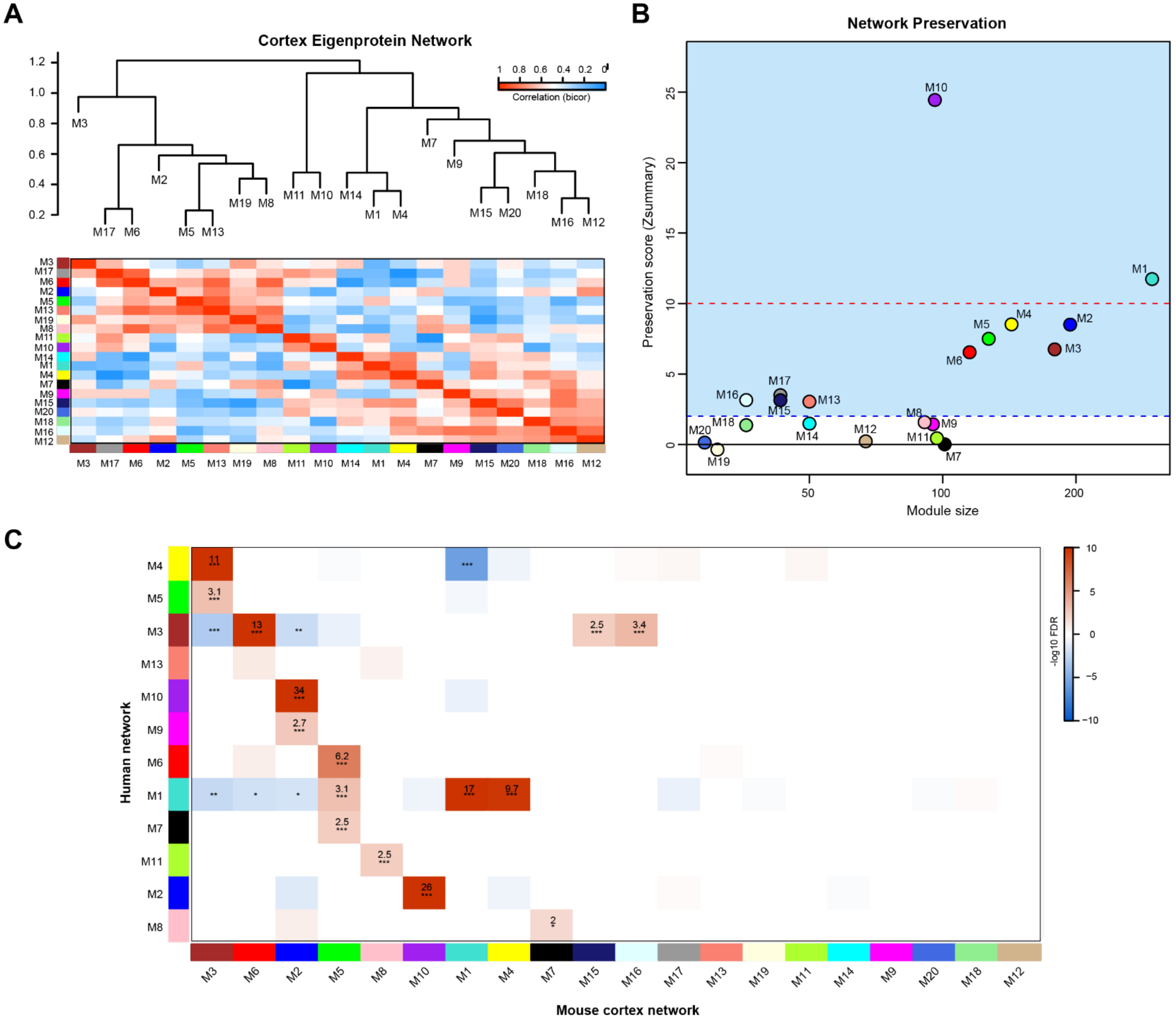

Supplementary Figure 4. **(A)** Eigenprotein network built using only cortex data showing module relatedness dendrogram and correlation (bicor). **(B)** Network preservation of mouse (cortex only) protein network in human frontal cortex network (Johnson et al., 2020). Modules with Zsummary greater than or equal to 1.96 (*q*=0.05, dashed blue line) are considered preserved, and modules with Zsummary of 10 or higher (*q*=1e-23, dashed red line) are considered highly preserved. The majority (11 out of 20) of consensus modules from the mouse (cortex only) proteome were found to be preserved in the human network. **(C)** Heatmap for the overrepresentation analysis (ORA) of mouse (cortex only) module members with human frontal cortex module members. Numbers in boxes are $-\log_{10}$ FDR values. *P* *<0.05, **<0.01, ***<0.001. Heatmap threshold is set at 10% FDR (0.1).

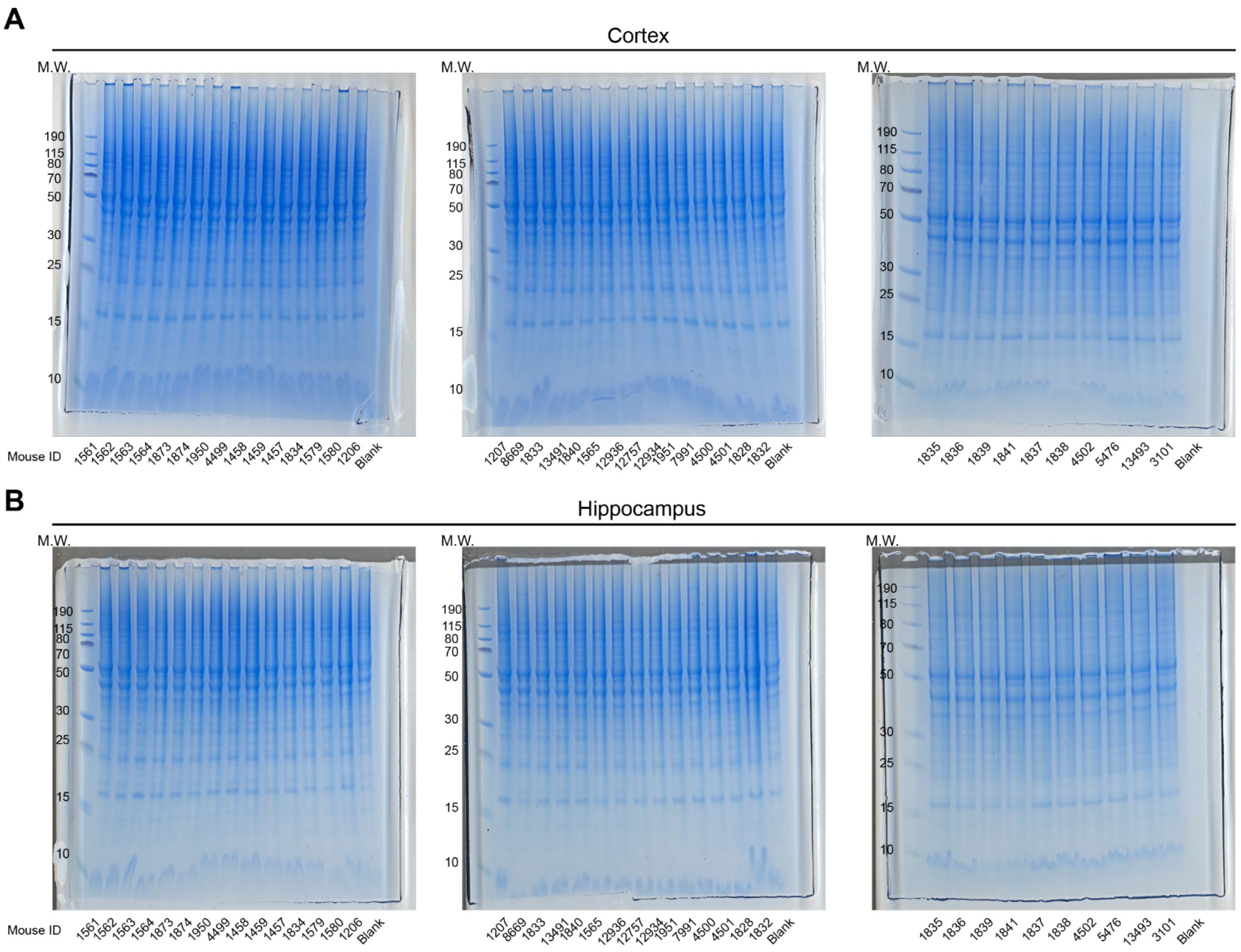

Supplementary Figure 5. (**A-B**) Protein integrity was assessed by SDS-PAGE gel stained with Coomassie blue in the cortex (**A**) and hippocampus (**B**).
